# An Exhibition of Caterers

**Published:** 1891-11-07

**Authors:** 


### AN EXHIBITION OF CATERERS.

The thirteenth annual exhibition of the Brewers, Mineral Waters, and allied trades was held last week at the Agricultural Hall, N. The number of exhibits was the largest yet shown. Some of them may be of interest to our readers. Among the rest, "The Twin Bottle and Bottling Company," of 101, Leadenhall Street, E.C., had a stand laden with small bottles containing claret, sherry, cider, &c., to each of which was attached a bottle of aërated water. Every bottle contains a quarter of a pint of wine, and its attached companion a like quantity of aërated water, both of which can be poured out separately or at the same time. The prices of these beverages are moderate, and they appear well worth a trial. Another exhibit of interest was that of Messrs. Crees and Co., of Warminster, Wilts, whose plate-washing, rinsing, racking, and drying machines would be most useful and economical in large institutions such as hospitals, asylums, infirmaries, &c., where a great deal of washing has to be done. The apparatus is capable of washing, rinsing, racking, and drying 3,000 plates per hour, and a boy is sufficient to work the machine. Among the disinfectants, a novelty known as Austin's Porous Disinfectant was shown. It consists of a cyclinder of porous earthenware filled with crystals of permanganate of potash hermetically sealed. When placed in water, the action of the liquid dissolves the crystals nearest the circumference, saturating the water with the percolating disinfectant. The disinfectant is manufactured by the Austin Sanitary Cylinder Company (Limited), 181, Queen Victoria Street, E.C.

An important exhibit was Messrs. Peterman's cockroach and beetle food and red ant destroyer. Our practical experience of this excellent article has been very satisfactory. Mr. Shorey, the proprietor, was kind enough to supply us with samples, which we have used and found most effectual in the destruction of these pests. The beetle and cockroach food is said to be non-poisonous to the larger animals. Samples and quantities may be obtained at 67, Farringdon Road, E.C.

